# Latent profile analysis of perceived vulnerability among parents of children with febrile seizures

**DOI:** 10.3389/fped.2025.1657584

**Published:** 2025-10-23

**Authors:** Yaxiu Cai, Haihong Zhu, Yanping Du, Xiaohong Wang, Cuie Chen, Yanping Jiang, Yuxiang Lan

**Affiliations:** ^1^ Respiratory Medicine Department, Yiwu Maternity and Children Hospital (Yiwu Hospital of Children’s Hospital Zhejiang University School of Medicine), Jinhua, China; ^2^ Nursing Department, The Children’s Hospital of Zhejiang University School of Medicine, National Clinical Research Center for Child Health, Hangzhou, China; ^3^ Nursing Department, Yiwu Maternity and Children Hospital (Yiwu Hospital of Children’s Hospital Zhejiang University School of Medicine), Jinhua, China; ^4^ Department of Neonatology, Yiwu Maternity and Children Hospital (Yiwu Hospital of Children's Hospital Zhejiang University School of Medicine), Jinhua, China; ^5^ Rehabilitation Department, Yiwu Maternity and Children Hospital (Yiwu Hospital of Children's Hospital Zhejiang University School of Medicine), Jinhua, China

**Keywords:** perceived vulnerability, influencing factors, latent profile analysis, febrile seizures, cross-sectional study

## Abstract

**Background:**

Certain parents of children with febrile seizures have a high sense of perceived vulnerability, which may lead to overprotective behaviors. This study aimed to measure the latent profile types of perceived vulnerability in parents of children with febrile seizures and investigate the factors affecting these different profiles.

**Methods:**

A cross-sectional study was conducted from October 2023 to December 2024. Participants were surveyed using a general data questionnaire, the child vulnerability scale (CVS), parents’ perception of uncertainty scale (PPUS), and perceived social support scale (PSSS). Latent profile analysis (LPA) was conducted to identify different types of perceived vulnerability among parents of children with febrile seizures. The influencing factors for each profile were identified using univariate and multivariate logistic regression analysis.

**Results:**

In total, 400 participants were included in this study. The perceived vulnerability among parents of children with febrile seizures was divided into three latent profiles: “General Low Perceived Vulnerability Group” (37.9%), “Moderate Perceived Vulnerability Group” (32.8%), and “High Perceived Vulnerability Group” (29.3%). Multivariate analysis indicated that relationship with children, parents’ age, educational attainment, marital status, body temperature during febrile seizures, PPUS, and PSSS were the factors affecting perceived vulnerability in parents of children with febrile seizures.

**Conclusion:**

The perceived vulnerability in parents of children with febrile seizures exhibited significant heterogeneity. To minimize the perceived vulnerability, medical professionals should provide tailored mental health counseling and intervention based on vulnerability characteristics.

## 1 Introduction

The International League Against Epilepsy (ILAE) defines febrile seizures as neurological diseases occurring in children aged between six months and five years, who were previously afebrile and experienced seizures associated with febrile diseases without an identifiable cause, such as neurodevelopmental infections, metabolic disorders, trauma, or intoxication (1). Febrile seizures is typically classified as simple febrile seizures and complex febrile seizures (2, 3). Simple febrile seizures account for 70%–80%, characterized by generalized seizures, no recurrence within 24 h, and no abnormal neurological signs (4). Unlike simple febrile seizures, complex febrile seizures occur less frequently but lead to more serious consequences. Complex febrile seizures account for 20%–30% of febrile seizures, with prolonged seizure duration or focal seizures, and recurrent attacks within 24 h, and can develop neurological disorders, such as temporal lobe epilepsy (5, 6). Febrile seizures are one of the most common reasons for admission to pediatric emergency departments worldwide and affect around 2%–5% of children (7). They affect approximately 3.9% of children in the United States and 7%–10% in Japan (8, 9). The prevalence rate of febrile seizures is 4.4% in China. Among individuals with febrile seizures, 22.7%–32% are predisposed to recurrent seizures, with complex febrile seizures increasing the risk of recurrence (10).

Although febrile seizures is typically regarded as a self-limiting condition with a generally benign nature (11, 12). The experiences associated with the diagnosis and treatment of febrile seizures and traumatic events can significantly shape parental approaches to child-rearing, often leading to severe mental problems (13). The prevalence of parents’ anxiety, stress, and depression was 58.2%, 29%, and 23.6% when their children were admitted for the treatment of febrile seizures in the hospital (13). The event may undermine the quality of family life, with parents experiencing prolonged anxiety and insecurity whenever their child develops a fever (14). In some cases, due to the lack of knowledge and understanding about febrile seizures, even 39% of mothers interpret the seizure as death, possibly because witnessing a febrile seizures is a distressing experience for parents (15). The symptoms and manifestations of febrile seizures are diverse and may include generalized or focal seizures, loss of consciousness, and facial or limb twitching (16). Furthermore, parents subjectively believe that their children are particularly prone to illness and suffer from a higher risk of death, which leads to psychological problems for themselves.

Parental perceptions of child vulnerability (PPCV) refers to the subjective conviction of parents whose children are especially prone to diseases and suffer from an increased risk of death. Generally, these children have a history of prior diseases but are in a healthy or stable state (17). A heightened sense of vulnerability among parents of children who have experienced febrile seizures may lead to their overprotective behaviors (18). Such behaviors can negatively affect the psychological and behavioral development of children, lead to the overuse of medical resources, and significantly damage families and society as a whole. Therefore, it is imperative to address the concern about perceived vulnerability among parents of children with febrile seizures. Nevertheless, few studies have concentrated on this issue. The current study on parents’ perceived vulnerability emphasizes aggregate scale scores, neglecting individual variations (19, 20). To address these deficiencies, latent profile analysis (LPA) presents a more suitable analytical framework. LPA is an individual-centric analytical approach that classifies samples based on diverse characteristics and performs analysis at the individual level (21, 22). It clarifies the connections between external continuous variables through the use of latent categorical variables. Thus, we employed latent profile analysis to identify the heterogeneity in perceived vulnerability among parents of children with febrile seizures. We explored the effects of sociodemographic attributes, disease-related factors, parental perceptions of uncertainty, and social support on perceived vulnerability.

The research questions of this study were as follows: (1) What categories can the perceived vulnerability of parents be divided into? (2) What are the characteristics of the perceived vulnerability of parents? (3) How do individual factors, such as sociodemographic characteristics and disease factors, parents’ perception of uncertainty, and social support, affect perceived vulnerability in different subgroups of parents?

## 2 Materials and methods

### 2.1 Study design and ethical considerations

This was a multi-center cross-sectional study. The survey was conducted in pediatric wards of three tertiary grade-A hospitals in Hangzhou and Jinhua, Zhejiang Province from October 2023 to December 2024. This study employed a convenience sampling method, enrolling parents of children with the onset of a feverish seizure who had visited the pediatric wards. Potential participants were directly approached the day following their child's admission to the pediatric ward. Following the acquisition of informed consent, participants completed the assessment measures with the assistance of researchers. To ensure confidentiality, data collection utilized code numbers exclusively. Approval of the study was obtained from the Ethics Committee of Yiwu Maternal and Child Health Hospital (2023-09-01). This study adhered to the STROBE guidelines (23).

### 2.2 Sample size estimation

The determination of sample size was based on Kendall's principle, considering the nature of quantitative cross-sectional studies, the sample size should be at least 5–10 times the number of independent variables (24). Considering 10% of invalid questionnaires, *n* = 14 × (5–10) + 10% × [14 × (5–10)] = (70–140) + (7–14) = 77–154. The final sample size should be greater than 77 participants. Finally, 400 sample sizes were included in this study.

### 2.3 Participants

The criteria were as follows: (1) parents whose children aged 6 months to 5 years old and diagnosed with febrile seizures according to ILAE classification, integrating clinical symptoms, electroencephalogram (EEG) data, blood test results, and cerebrospinal fluid examination findings (25); (2) the parents had normal cognition and voluntarily participated in this study. Exclusion criteria: (1) children with chronic, neurological, psychiatric illnesses, nervous system infections, or genetic diseases. (2) seizures caused by organic, metabolic, or abnormal conditions; (3) medical history with other current or previous illnesses for which the children were hospitalized, underwent chronic drug therapy, growth and developmental delays or prematurity; (4) parents with a history of mental illness such as anxiety and depression.

### 2.4 Measures

Data collection was conducted in the pediatric ward, involving face-to-face interactions by five trained nurses.

#### 2.4.1 General data questionnaire

The questionnaire was developed by the research team based on a comprehensive review of literature, including social demographic data (such as age of parents, sex, educational attainment, and marital status) as well as disease-related information (whether it is the first episode, the duration of the seizure, and the body temperature at the time of seizure onset).

#### 2.4.2 Child vulnerability scale (CVS)

The child vulnerability scale, developed by Forsyth et al. (26) was used to assess parents’ concerns about their children's health. Its Chinese version was translated and validated by Yuan (27) with Cronbach's α coefficient of 0.791 for confrontation. This scale consists of two dimensions, namely the actual disease condition of the child (5 items) and the parents’ fear of losing the child (3 items). A four-point Likert scale from 0 (completely disagree) to 3 (completely agree) was used to score 0–24. The total score ranges from 0 to 24 points. The higher the score, the higher the level of perceived vulnerability of the parents. A total score of ≥10 score indicates a high level of perceived vulnerability, and total <10 score indicates general level. In the present study, the overall Cronbach's alpha coefficient of this scale was 0.899.

#### 2.4.3 Parents’ perception of uncertainty scale (PPUS)

This scale was developed by Mishel (28). The Chinese version was translated by Mai et al. (29) with the scale demonstrating a Cronbach's α coefficient of 0.928. The scale has 31 items over 4 dimensions: uncertainty (13 items), complexity (9 items), information deficiency (5 items), and unpredictability (4 items). Each item is scored from 1 to 5 (strongly disagree, disagree, uncertain, agree, and strongly agree), with a total score range of 31–155. A higher score indicates a higher level of disease uncertainty. In this study, Cronbach's α of the scale was 0.938.

#### 2.4.4 Perceived social support scale, PSSS

This scale was formulated by Zimet et al. (30). Jiang (31) translated this scale into Chinese with the scale demonstrating a Cronbach's α coefficient of 0.899. It was primarily employed to assess the extent of social support that an individual comprehends or senses from various groups of people. This scale is divided into three dimensions: family support(4 items), friend support(4 items), and other support(4 items), with a total of 12 items. Each item is scored using a 7-point Likert scale, with a total score ranging from 12 to 84. A score of 12–36 indicates low support, 37–60 indicates moderate support, and 61–84 indicates high support. In this study, Cronbach's α of the scale was 0.932.

## 3 Data analysis

Profile classifications of perceived vulnerability for participants were identified using Mplus 8.0 software. The Pearson chi-square test, likelihood ratio chi-square test, Akaike information criterion (AIC), Bayesian information criterion (BIC), and sample size-adjusted BIC were utilized to examine the discrepancies between expected and observed values, thereby assessing the model's goodness-of-fit. Lower values indicate a superior match. The bootstrap likelihood ratio test (BLRT) and likelihood ratio test (Lo-Mendell-Rubin, LMR) were utilized to contrast the fit differences among various models. The closer the entropy value is to 1, the more accurate the classification.

The best classification model for the perceived vulnerability of parents of children with febrile seizures was determined based on the results of latent profile analysis. The general demographic data and disease factors of parents in different categories of perceived vulnerability were compared using the chi-square test, Kruskal–Wallis rank sum test or univariate analysis with SPSS 26.0 statistical software. Multinominal logistic regression was used to take the latent categories of parents’ perceived vulnerability as the dependent variable and the factors with significant differences in the univariate analysis as the independent variables to further explore the influencing factors of parents in different latent categories of perceived vulnerability. A *P*-value < 0.05 was considered statistically significant.

## 4 Results

### 4.1 Characteristics of parents of children with febrile seizures

The demographic and clinical information, as well as the scores of parents’ perception of uncertainty and perceived social support of the research subjects, were presented in Table 1.

**Table 1:** Sociodemographic characteristics of research subjects and inter group comparisons.

Characteristics	Category	Classification of latent profiles			Overall (*n* = 400) [%]	*F/χ^2^/H*	*P*
		Class 1 [%] (*n* = 152)	Class 2 [%] (*n* = 131)	Class 3 [%] (*n* = 117)			
Relationship with children	Father	81 (53.29)	34 (25.95)	45 (38.46)	160 (40.00)	22.069^b^	<0.001
	Mother	71 (46.71)	97 (74.05)	72 (61.54)	240 (60.00)		
Parents’ age(years)	≤30	8 (5.26)	63 (48.09)	16 (13.68)	87 (21.75)	134.184^b^	<0.001
	30–39	131 (86.19)	60 (45.80)	57 (48.72)	248 (62.00)		
	≥40	13 (8.55)	8 (6.11)	44 (37.60)	65 (16.25)		
Educational attainment	Undergraduate or above	75 (49.34)	25 (19.08)	24 (20.51)	124 (31.00)	26.150^c^	<0.001
	College degree	31 (20.39)	35 (26.72)	37 (31.62)	103 (25.75)		
	Senior high school	29 (19.09)	40 (30.54)	29 (24.79)	98 (24.50)		
	Junior middle school and below	17 (11.18)	31 (23.66)	27 (23.08)	75 (18.75)		
Marital status	Married	136 (89.47)	99 (75.57)	89 (76.07)	324 (81.00)	11.448^b^	0.003
	Unmarried/divorced/widowed	16 (10.53)	32 (24.43)	28 (23.93)	76 (19.00)		
Monthly family income(RMB)	<1,000	18 (11.84)	19 (14.50)	18 (15.39)	55 (13.75)	2.021^c^	0.364
	1,000–2,999	36 (23.68)	32 (24.43)	28 (23.93)	96 (24.00)		
	3,000–5,000	59 (38.82)	47 (35.88)	52 (44.44)	158 (39.50)		
	>5,000	39 (25.66)	33 (25.19)	19 (16.24)	91 (22.75)		
Children's gender	Female	76 (50.00)	66 (50.38)	63 (53.85)	205 (51.25)	0.450^b^	0.798
	Male	76 (50.00)	65 (49.62)	54 (46.15)	195 (48.75)		
Children's age(years)	≤1	59 (38.82)	38 (29.01)	41 (35.04)	138 (34.50)	3.610^b^	0.461
	2–3	52 (34.21)	54 (41.22)	47 (40.17)	153 (38.25)		
	4–5	41 (26.97)	39 (29.77)	29 (24.79)	109 (27.25)		
Only child	No	59 (38.82)	48 (36.64)	50 (42.74)	157 (39.25)	0.982^b^	0.612
	Yes	93 (61.18)	83 (63.36)	67 (57.26)	243 (60.75)		
Primary onset	No	67 (44.08)	54 (41.22)	46 (39.32)	167 (41.75)	0.639^b^	0.727
	Yes	85 (55.92)	77 (58.78)	71 (60.68)	233 (58.25)		
Body temperature during febrile seizures(°C)	≤38.0	8 (5.27)	7 (5.34)	15 (12.82)	30 (7.50)	40.398^c^	<0.001
	38.1–39.0	9 (5.92)	13 (9.93)	48 (41.03)	70 (17.50)		
	39.1–41.0	112 (73.68)	65 (49.62)	33 (28.20)	210 (52.50)		
	>41.0	23 (15.13)	46 (35.11)	21 (17.95)	90 (22.50)		
Number of febrile seizures occurrences(times)	1	68 (44.74)	57 (43.51)	55 (47.01)	180 (45.00)	4.301^b^	0.636
	2	55 (36.18)	54 (41.22)	41 (35.04)	150 (37.50)		
	3	23 (15.13)	16 (12.21)	20 (17.09)	59 (14.75)		
	≥4	6 (3.95)	4 (3.05)	1 (0.85)	11 (2.75)		
Duration of febrile seizures(minutes)		2.64 ± 1.71	2.56 ± 1.57	2.73 ± 1.52	2.64 ± 1.61	0.311^a^	0.733
PPUS		84.61 ± 12.88	97.98 ± 13.58	105.37 ± 13.87	95.06 ± 15.95	83.905^a^	<0.001
PSSS		54.38 ± 14.35	52.02 ± 14.43	36.48 ± 16.69	48.37 ± 16.92	52.180^a^	<0.001

Body temperature during febrile seizures was measured at peak of seizure onset, measurement method is tympanic. Classification basis: Low fever 37.5–38°C, moderate fever: 38.1–39.0°C, high fever: 39.1–41.0°C, hyperpyrexia: >41.0°C.

^a^ F.

^b^ *χ^2^.*

^c^ *H*.

### 4.2 Latent profile analysis of perceived vulnerability among parents of children with febrile seizures

A latent profile analysis was performed on the CVS scores of 400 research participants, utilizing the scores of its eight items as manifest indicators. Latent profile models ranging from one to four categories were sequentially fitted, beginning with the baseline model comprising a single category, as detailed in Table 2. Among these models, the three-category model exhibited the moderate substantial reduction in AIC, BIC, and aBIC values, achieved an entropy value of 0.921 (exceeding the threshold of 0.900), and yielded *P*-values of less than 0.05 for both the LMR and BLRT tests. In view of the non-significant LMR for Model 4, and considering parsimony and clinical interpretability, the three-category model was consequently selected as the optimal representation for the perceived vulnerability categories among parents of children with febrile seizures. To validate the accuracy of this optimal latent profile analysis model, discriminant analysis was employed. The average posterior probability (AvePPs) were 0.954, 0.991, and 0.958 for the three classes, all well above the recommended threshold of.70, indicating excellent classification certainty. The OCC values were 20.739, 110.111, and 22.810, respectively, far exceeding the commonly accepted cutoff of 5, suggesting highly accurate class assignment. Inspection of bivariate residuals (TECH10) showed no substantial violations of local independence (all|z| < 1.96).

**Table 2:** Fit statistics for each profile structure.

Model	k	AIC	BIC	aBIC	Entropy	*P*		Probability of class
						LMR	BLRT	
1	16	7,118.631	7,182.495	7,131.726	–	–	–	–
2	25	5,745.224	5,845.01	5,765.684	0.908	<0.001	<0.001	0.43861/0.56139
3^a^	34	5,451.1	5,586.81	5,478.926	0.921	<0.001	<0.001	0.37905/0.32827/0.29268
4	43	5,399.881	5,571.514	5,435.073	0.943	0.1016	<0.001	0.36734/0.13844/0.19949/0.29473

AIC, Akaike information criterion; BIC, Bayesian information criterion; aBIC, the sample size adjusted Bayesian information criterion; LMR, *P* value for the Lo-Mendell-Rubin; BLRT, *P* value for the Bootstrapped Likelihood Ratio Test.

^a^ Optimal mode. In view of the non-significant LMR for Model4, and consider parsimony and clinical interpretability, Model 3 was ultimately chosen.

**Figure 1:**
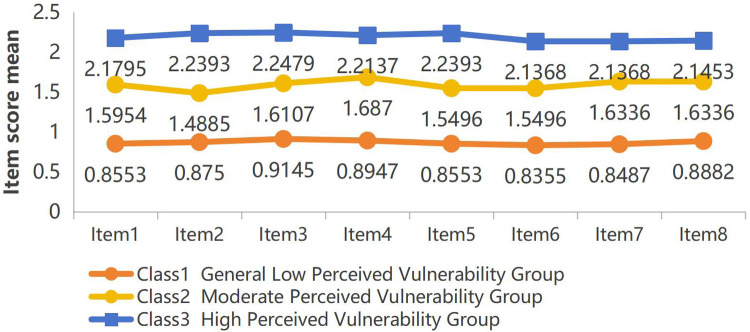
Distribution of three-category latent profile analysis of perceived vulnerability among parents of children with febrile seizures(actual disease condition of the child dimension:item 1, 2, 3, 5, 8, parents’ fear of losing the child dimension:item 4, 6, 7).

According to the results of latent profile analysis, class 1 accounted for 37.9% (152 cases), class 2 accounted for 32.8% (131 cases), and class 3 accounted for 29.3% (117 cases). The characteristics of the three latent categories of perceived vulnerability among parents of children with febrile seizures were analyzed by drawing the line graphs of the scores of each item of the CVS (Figure 1). The characteristics of each category were named based on the fluctuation of the mean line graphs of each item. Class 1 was named “General Low Perceived Vulnerability Group”, class 2 was named “Moderate Perceived Vulnerability Group”, and class 3 was named “High Perceived Vulnerability Group”.

### 4.3 Analysis of perceived vulnerability of latent profile

Table 3 shows the mean scores of eight items and total scores of CVS. The scores of each item and the total score of Class 1 are all lower than those of the other categories. And the total score of Class 1 was less than 10 scores, indicating that its perceived vulnerability was at a general level. The total score of Class 2 and class 3 was not less than 10 scores, indicating that their perceived vulnerability were at a high level.

**Table 3:** Scores of relevant items and total scores of the three category perceived vulnerability among parents of children with febrile seizures(`$\bar{X}\pm S$).

Item	General low perceived vulnerability group(class 1)	Moderate perceived vulnerability group(class 2)	High perceived vulnerability group(class 3)
1. My child gets more colds than other children I know.	0.86 ± 0.47	1.59 ± 0.57	2.18 ± 0.45
2. I often think about calling the doctor about my child.	0.88 ± 0.48	1.49 ± 0.57	2.24 ± 0.47
3. When there is something going around my child usually catches it.	0.91 ± 0.43	1.61 ± 0.55	2.25 ± 0.47
4. In general my child seems less healthy than other children.	0.89 ± 0.46	1.56 ± 0.61	2.21 ± 0.43
5. Often have to keep my child indoors because of health reasons.	0.86 ± 0.44	1.64 ± 0.56	2.24 ± 0.45
6. Sometimes I get concerned that my child doesn't look as healthy as s/he should.	0.84 ± 0.45	1.69 ± 0.58	2.14 ± 0.41
7. I get concerned about circles under my child's eyes.	0.85 ± 0.49	1.56 ± 0.56	2.14 ± 0.51
8. I often check on my child at night to make sure s/he is okay.	0.89 ± 0.42	1.63 ± 0.54	2.15 ± 0.42
Total average score	6.97 ± 1.06	12.75 ± 1.20	17.54 ± 1.20

### 4.4 Univariate analysis of perceived vulnerability among parents of children with febrile seizures

The results of univariate analysis showed statistically significant (*P* < 0.05) between-group differences in relationship with children (*x*^2^ = 22.069, *P* *<* 0.001), parents’ age (*x*^2^ = 134.184, *P* *<* 0.001), educational attainment (*H*=26.150, *P* *<* 0.001), marital status (*x*^2^ = 11.448, *P* *=* 0.003), body temperature during febrile seizures (*H*=40.398, *P* *<* 0.001), PPUS (*x*^2^ = 83.905, *P* *<* 0.001), and PSSS (*x*^2^ = 52.180, *P* *<* 0.001). The aforementioned factors may influence the perceived vulnerability among parents of children with febrile seizures. Detailed information was shown in Table 1.

### 4.5 Multifactor analysis of perceived vulnerability among parents of children with febrile seizures

Using Class 1 as the reference group, a multiple logistic regression was performed on factors that showed statistically significant differences in univariate analysis to determine predictive factors associated with the perceived vulnerability among parents of children with FS. The assignment of dependent and independent variables was shown in Table 1. The predictive factors for the perceived vulnerability among parents of children with febrile seizures are shown in Table 4, including relationship with children, marital status, parents’ age, educational attainment, body temperature during febrile seizures, PPUS, and PSSS.

**Table 4:** Results of multivariate regressions predicting perceived vulnerability.

Items	Class 2 vs. class 1			Class 3 vs. class 1		
	*P*	OR	OR 95% CI	*P*	OR	OR 95% CI
Relationship with children						
Mother	0.000	4.398	2.027–9.541	0.000	5.735	2.292–14.351
Marital status						
Unmarried/divorced/widowed	0.017	3.659	1.264–10.590	0.009	4.795	1.469–15.647
Parents’ age						
≤30	0.000	45.027	7.019–288.867	0.835	1.218	0.190–7.830
30–39	0.595	0.655	0.137–3.117	0.001	0.088	0.020–0.386
Educational attainment						
Junior middle school or below	0.007	3.759	1.440–9.812	0.013	4.042	1.336–12.225
Senior high school	0.000	9.900	3.149–31.128	0.000	11.563	3.186–41.964
College degree	0.004	4.260	1.605–11.308	0.394	1.661	0.518–5.329
Body temperature during febrile seizures						
≤38.0°C	0.231	3.511	0.451–27.352	0.285	3.231	0.376–27.727
38.1∼39.0°C	0.081	0.264	0.059–1.176	0.238	2.478	0.549–11.185
39.1∼41.0°C	0.001	0.182	0.064–0.514	0.000	0.093	0.027–0.316
PPUS	0.000	1.159	1.114–1.206	0.000	1.177	1.128–1.228
PSSS	0.021	0.971	0.947–0.996	0.000	0.908	0.883–0.935

Compared with Class 1, mothers and marital status(single, divorced, or widowed), were more likely to be classified as Class 3. Meanwhile, higher PPUS and lower PSSS traits were more likely to be classified as Class 3. Compared with parents aged 40 and above, those aged 30–39 have a reduced risk of experiencing perceived vulnerability (OR = 0.088, 95% CI 0.020–0.386, *P* = 0.001). Individuals with an educational level of junior high school or below (OR = 4.042, 95% CI 1.336–12.225, *P* = 0.013) and high school (OR = 11.563, 95% CI 3.186–41.964, *P* = 0.000) had an increased risk of high-level perceived vulnerability. The probability of increased perceived vulnerability among parents with children's body temperature 39.0–41.0°C during FS was 0.093 times that of other age groups (OR = 0.093, 95% CI 0.027–0.316, *P* = 0.000). Compared with Class 1, Parents who are mothers, age (years) ≤30, marital status unmarried/divorced/widowed, lower educational attainment, body temperature 39.0–41.0°C during FS, higher PPUS, and lower PSSS were more likely to be classified as Class 2. The probability of increased perceived vulnerability among mothers was 4.398 times that of other groups (OR = 4.398, 95% CI 2.027–9.541, *P* = 0.000). The probability of increased perceived vulnerability among unmarried/divorced/widowed parents was 3.659 times that of other groups (OR = 3.659, 95% CI 1.264–10.590, *P* = 0.017). The probability of increased perceived vulnerability among parents aged ≤30 was 45.027 times that of other groups (OR = 45.027, 95% CI 7.019–288.867, *P* = 0.000). As the educational level decreases, the perceived vulnerability among parents tends to increase. A body temperature between 39.0 and 41.0°C significantly decreased the risk of perceived vulnerability (OR = 0.182, 95% CI 0.064–0.514, *P* = 0.001). The higher the score of PPUS, the higher the level of parents’ perceived vulnerability, which is statistically significant (OR = 1.159, 95% CI 1.114–1.206, *P* = 0.000). The lower the score of PSSS, the higher the level of parents’ perceived vulnerability, which is statistically significant (OR = 0.971, 95% CI 0.947–0.996, *P* = 0.021).

## 5 Discussion

Our findings revealed substantial heterogeneity in perceived vulnerability among parents of children with febrile seizures, highlighting the necessity for tailored intervention. Similar to previous studies, participants were categorized into three distinct groups, including “Low Perceived Vulnerability Group” (37.9%), “Moderate Perceived Vulnerability Group” (32.8%), and “High Perceived Vulnerability Group” (29.3%) based on their characteristics (32). Nevertheless, in certain studies on parents of children with chronic diseases, the participants were classified into two or four groups (33, 34). The differences in parental response are related to the disease population, study objective, sample size, and disease symptoms (35, 36). Given that non-significant LMR for other classifications were not significant, and considering parsimony and clinical interpretability, three subgroups were most suitable for this study.

Participants in class 1 accounted for 37.9% of the participants. Additionally, the level of perceived vulnerability in class 1 was at a general level, with a total CVS score of <10 and an average item score of <1. This is different from the results of Wang et al. (32). The reason might be that our questionnaires were distributed among parents on the next day of admission. At this point, the children's conditions were more stable, and parents’ emotions partly improved. The majority (62.1%) of parents of children with febrile seizures were classified as class 2 (Moderate Perceived Vulnerability Group) and class 3 (High Perceived Vulnerability Group), with a total CVS score of ≥10, indicating that the perceived vulnerability among parents was at a high level. This result is similar to that of Othman et al. (13), and can possibly be explained by the nature of the disease. Although febrile seizures are classified as benign conditions, 91% of parents reported severe anxiety after witnessing the first febrile seizure (14). Moreover, based on studies on patients with epilepsy, a significant association exists between parental stress, emotional and behavioral symptoms of children (37, 38). Class 3 consisted of the smallest proportion of patients but had the highest perceived vulnerability. Liu et al. (36) conducted an LPA and discovered three potential parental burnout profiles in parents of children with autism spectrum disorder. The high parental burnout profiles (8%) accounted for the smallest proportion but had the highest perceived vulnerability, which is similar to our findings. It is of great importance to promptly identify individuals with high perceived vulnerability and provide timely interventions.

It is essential for healthcare providers to acknowledge the varying care requirements of parents across different categories and to swiftly identify and deliver timely psychological support. Healthcare providers should prioritize interventions for parents with higher levels of perceived vulnerability and deliver tailored strategies based on the unique characteristics of each group. For those in the High Perceived Vulnerability Group who experienced severe mental stress, emotional and behavioral problems, intensive psychological counseling, timely medical treatment, and health education are essential to bolster hope and counteract negative disease perceptions. Peer-led support can alleviate perceived vulnerability for the Moderate Perceived Vulnerability Group. In addition, it is necessary to disseminate knowledge regarding the methods of physical antipyretic measures and pharmacologically-induced temperature reduction. For the Low Perceived Vulnerability Group, healthcare providers should maintain a positive mental state through regular health and medical interventions.

The results showed that mothers were more likely to experience a higher level of perceived vulnerability. The reasons are as follows: Firstly, mothers with affected children possess low febrile seizure knowledge and exhibit a high level of anxiety and uncertainty (39, 40). Studies have shown that perceived vulnerability is significantly associated with disease uncertainty and anxiety (41). Secondly, from a physiological standpoint, mothers can resonate with their children's emotional states (42). Studies have revealed that when mothers observe their children in stressful situations, the variations in their facial temperatures exhibit synchrony with those of their children (43, 44). Thirdly, during the process of parenting, mothers typically show a high level of attentiveness and sense of responsibility (45). This emotional engagement renders them more susceptible to their own vulnerability. Therefore, it is necessary to strengthen psychological counseling for this group of mothers, eliminate their inner concerns as much as possible, and explain in detail the child's condition and the progress of treatment to enhance mothers’ confidence.

Marital status affects parents’ perceived vulnerability, which is consistent with the results of Moncrief et al. (46). Single parents receive less social support and have poorer family functions, which makes them prone to negative emotions and leads to a lack of confidence in disease management and health care.

Educational attainment can also affect parents’ perceived vulnerability. Parents with low educational levels may feel stressed and anxious when taking care of a sick child due to a lack of sufficient health knowledge and resources (47). In addition, parents with low educational levels may encounter challenges in accurately assessing the severity of their children's disease and determining the appropriate timing for seeking medical assistance, resulting in unwarranted visits to emergency departments (48).

Age also affects parents’ perceived vulnerability. Parents’ age ≤30 increases the risk of perceived vulnerability, but an age range of 30–39 decreases the risk of perceived vulnerability. Young parents have less life experience and poorer psychological resilience. The lack of life experience and parenting knowledge may bring more difficulties and challenges in the parenting process (49). Therefore, supporting young parents in their parenting journey requires not only providing practical parenting knowledge and skills but also offering emotional support and encouragement. A body temperature between 39.0 and 41.0°C significantly decreased the risk of perceived vulnerability in this study. When a child suffers from a high body temperature, parents tend to think that the seizures are caused by the high temperature rather than other neurological disorders. However, the conclusions should be interpreted cautiously due to the cross-sectional design and potential measurement/contextual biases in temperature ascertainment.

The results of multifactor analysis showed that parents’ perception of uncertainty and perceived social support were significantly correlated with the perceived vulnerability of parents of children with febrile seizures. A more PPUS was associated with a higher parents’ perceived vulnerability. This study showed that compared to class 1, parents with higher PPUS were more likely to be classified as class 2. This is similar to the results of Mullins et al. (50). Disease uncertainty refers to the cognitive experience of an unclear disease state and a loss of control over prognosis due to the lack of relevant information or clues (51). For parents, their child's illness is a very serious and stressful event. A higher sense of disease uncertainty may lead to cognitive deficiencies and confusion regarding their child's condition, treatment methods, and prognosis when facing their child's disease (52, 53). This confusion may lead to their extreme worry and helplessness, leading to a sense of perceived vulnerability. Nurses should pay attention to the disease uncertainty of parents of children with febrile seizures. They can help parents of children to timely and accurately grasp the information about their children's condition by providing relevant information and psychological support, thereby reducing the disease uncertainty and perceived vulnerability.

Perceived social support refers to an individual's belief or evaluation regarding the extent to which their social networks, such as family and friends, offer informational, physical, or psychological assistance (54). This study showed that compared to class 1, parents with low perceived social support were more likely to belong to class 2. This indicated that parents of children with febrile seizures who had a high level of perceived social support were more likely to feel emotionally fulfilled and secure, alleviating their worries and anxiety, enhancing their ability to cope with the disease, and reducing their sense of vulnerability. This finding was consistent with those reported by previous studies (55). Therefore, medical staff should focus on enhancing the perceived social support of parents of children with febrile seizures, assist in mobilizing their social support systems, actively provide psychological counseling, encourage them to express and share their emotions, and actively seek external support to enhance perceived social support and reduce perceived vulnerability.

## 6 Study strengthens and implications

This study utilized LPA to categorize the perceived vulnerability levels of parents of children with febrile seizures and investigated the factors affecting perceived vulnerability. LPA can offer more detailed and personalized insights into variable interrelationships, which can help formulate more feasible and effective strategies to accommodate the unique needs and preferences of different populations (56). The results indicated that the perceived vulnerability of parents of children with febrile seizures deserves more attention from society and the public.

By identifying the characteristic subgroups of perceived vulnerability among parents of children with febrile seizures, this study provided a foundation for developing targeted intervention measures that can leverage the advantages of these personality traits to enhance psychological resilience and prevent the progression of perceived vulnerability. Secondly, this study clarified the potential categories of perceived vulnerability among parents of children with febrile seizures and identified the factors associated with each subgroup. These findings can help improve prevention strategies. By identifying the modifiable risk factors associated with specific subgroups, including relationship with children, parents’ age, educational attainment, marital status, body temperature during febrile seizures, PPUS, and PSSS, healthcare providers can offer more effective and personalized care plans and modify the external factors to mitigate their impact on perceived vulnerability. Finally, further validation of interventional measures based on parents’ perceived vulnerability is of great importance. Specifically, studies should focus on how parents with different degrees of perceived vulnerability respond to various intervention measures to determine which interventional measures are most effective in improving parents’ perceived vulnerability. Additionally, studies should investigate how these personality-based interventions can be seamlessly integrated into parents’ daily care practices for their children.

## 7 Limitations

Firstly, the cross-sectional design employed in this study precludes the possibility of establishing causal inferences. Secondly, convenience sampling has poor representativeness and is prone to selection bias. Thirdly, only the parents in the inpatient department were surveyed. The absence of a normal control group (parents of children without febrile seizures) makes it impossible to reflect the particular characteristics of the parents of children with such diseases. Hospital clustering also existed in this study. In addition, the samples were selected solely from Zhejiang Province, which may have restricted the generalizability of our findings. Future studies should expand the sample size and use cross-cultural adaptations. Moreover, we did not include different clinical subtypes of febrile seizure and care pathway variables. In future studies, simple and complex febrile seizures, first and recurrent events, and time from seizure to survey should be included in regression models. Even though statistical analysis confirmed the absence of methodological biases, reporting biases may persist, potentially undermining the accuracy of the results.

## 8 Conclusion

In conclusion, using latent profile analysis, the study classified perceived vulnerability into three distinct groups: the Low Perceived Vulnerability Group, the Moderate Perceived Vulnerability Group, and the High Perceived Vulnerability Group. The relationship with children, parents’ age, educational attainment, marital status, body temperature during febrile seizures, PPUS, and PSSS affected the perceived vulnerability of parents of children with febrile seizures. Medical staff should provide targeted mental health intervention based on the perceived vulnerability characteristics to reduce the perceived vulnerability of parents of children with febrile seizures.

## Data Availability

The original contributions presented in the study are included in the article/Supplementary Material, further inquiries can be directed to the corresponding author.
